# The Role of the Gut Microbiota on the Beneficial Effects of Ketogenic Diets

**DOI:** 10.3390/nu14010191

**Published:** 2021-12-31

**Authors:** Ilias Attaye, Sophie van Oppenraaij, Moritz V. Warmbrunn, Max Nieuwdorp

**Affiliations:** Department of Internal Medicine and Vascular Medicine, Amsterdam University Medical Center, Location Academic Medical Center, 1105 Amsterdam, The Netherlands; sophievoppenraaij@gmail.com (S.v.O.); m.v.warmbrunn@amsterdamumc.nl (M.V.W.)

**Keywords:** diet, ketogenic diet, gut microbiota, seizure, cardiometabolic disease

## Abstract

The ketogenic diet is a dietary regime focused on strongly reducing carbohydrate intake and increasing fat intake; leading to a state of ketosis. The ketogenic diet has gained much popularity over the years due to its effects on promoting weight loss, increasing insulin sensitivity and reducing dyslipidaemia. All these factors play a crucial role in the development of cardio-metabolic diseases; one of the greatest health challenges of the time. Moreover, the ketogenic diet has been known to reduce (epileptic) seizure activity. It is still poorly understood how following a ketogenic diet can lead to these beneficial metabolic effects. However, in recent years it has become clear that diet and the gut microbiota interact with one another and thus influence host health. The goal of this review is to summarize the current state of knowledge regarding the beneficial metabolic effects of the ketogenic diet and the role of gut microbiota in these effects.

## 1. Introduction

The ketogenic diet is a form of diet focusing on reduced intake of carbohydrates and increased intake of fat leading to a state of ketosis [1]. Different protocols exist of ketogenic diets that differ in caloric content and macronutrient percentage which find applications in neurological and metabolic disorders. Specifically, the classic ketogenic diet (cKD) has been used to treat epilepsy in children continuously since 1921. cKD is a normocaloric high-fat very low-carbohydrate diet, used worldwide for the treatment of drug-resistant epilepsy (DRE) for its anticonvulsant effect and is typically composed of a 4:1 ratio of fat (in grams) to protein plus carbohydrates (in grams), thus shifting the predominant caloric source from carbohydrates to fat [2]. However, there are variations of this diet in which this fat to protein and carbohydrate ratio differs (e.g., modified Atkins diet, low glycemic index treatment, and medium-chain triglyceride diet) [2,3]. Another variation is the very low-calorie ketogenic diet (VLCKD) has been recently proposed as an appealing nutritional strategy for obesity management. The VLCKD is characterized by a low carbohydrate content (<50 g/day), 1–1.5 g of protein/kg of ideal body weight, 15–30 g of fat/day, and a daily intake of about 500–800 calories [4].

In recent years the ketogenic diet has gained much of its former position back as a potential method to promote weight loss and reduce insulin resistance in both type 1 and type 2 diabetes [5,6,7]. However, concerns also exist about the long-term efficacy in weight loss and the potential adverse effects on renal function and blood lipid levels due to increased intake of protein and fat [7]. Especially in diabetes, increased animal protein, but not plant protein intake has been associated with insulin resistance and increased morbidity [8,9]. Mechanisms of these results are largely unknown. However, a possible mechanism might be that the regulation of glucose and insulin levels could be undermined due to phosphorylation that is caused by branched-chain and aromatic amino acids, which are mainly derived from animal protein [9,10,11,12]. In addition, foods high in animal protein also contain other nutrients (e.g., saturated fat, haem iron, and nitrites) that might attribute to these results [13]. Moreover, diets high in animal protein are linked to gut microbially produced metabolites that are associated with insulin resistance and cardiovascular morbidity [14,15].

The exact mechanism by which a ketogenic diet promotes its beneficial metabolic effects regarding seizure activity, obesity, dyslipidaemia, and insulin resistance remains unknown, but recent evidence points towards a crucial role for the gut microbiota [16,17,18]. The gut microbiota is a relatively new discovered “endocrine organ” that has been associated with many metabolic conditions and has been shown to be strongly regulated by diet [14,19,20]. A systematic search (Supplementary Table S1) was carried out in PubMed, Medline and Embase in order to summarize the current state of knowledge regarding the metabolic effects of a ketogenic diet and the role of gut microbiota in these effects. Throughout this review we describe the dietary composition of the studied diets by defining the fat: protein + carbohydrate ratio.

## 2. The Gut Microbiota and Ketogenic Diet: Epilepsy

One of the first successful implementations of the ketogenic diet were its positive effects on refractory seizures [21]. A landmark observational study which was published in 1998 had shown that children with refractory seizures responded extremely well to the ketogenic diet (4:1), with a reduction of more than 50% of seizure activity already three months after initiation of diet. However, not all subjects responded to the diet, and it was difficult to maintain for extended periods [22]. The ketogenic diet is also the preferred treatment in subjects that suffer from Glucose Transporter-1 Deficiency syndrome (GLUT1-DS), a rare genetic mutation resulting in reduced glucose transport to the brain and subsequent seizures [23].

The exact mechanism by which a ketogenic diet contributes to reduced seizure activity remains unknown. However, two mouse studies have shown that gut microbiota composition and function are implicated to be involved in the seizure-reducing effects of a ketogenic diet (6:1 and 3:1) [18,24]. Rodent studies in rats have shown that transplanting faeces from stressed rats to naïve rats worsened epileptic seizure duration and intensity. This phenomenon was reversed when faeces were transplanted from naïve rats to stressed rats, indicating a crucial role for the gut microbiota [24]. Two other studies in mice shed light on mechanisms by which the gut microbiota is involved in the anti-seizure effects of a ketogenic diet (6:1 and 4:1) [18,25]. Mice that were fed a ketogenic diet (6:1) were less susceptible to seizures compared to the control group in the study of Olson et al. (2018) [18]. The protective effects of the ketogenic diet were reduced after a broad-spectrum antibiotic course but re-established after recolonizing with bacteria. The authors also found that a ketogenic diet reduced overall alpha diversity, while increasing the relative abundance of *Akkermansia muciniphila*. This is of note, since a reduced alpha diversity is generally associated with worsened metabolic outcomes [26], whereas high abundance of *A. muciniphila,* a known short-chain fatty acid (SCFA) producer, is associated with improved metabolic health [27,28]. SCFAs are microbially produced metabolites that are implicated in metabolic health. The most commonly studied SCFA are propionate, acetate and butyrate, and these are mainly produced from gut microbial fermentation of fibres [29]. Increased levels of SCFA have been implicated in lower levels of obesity and higher levels of insulin sensitivity [30,31], and it is currently thought that the beneficial effects of *A. muciniphila* following a ketogenic diet are exerted by changes in plasma levels of SCFAs [17]. Importantly, the results of the aforementioned study were not confounded by weight changes, as the ketogenic diet treated mice had similar weight distributions compared to the control diet treated mice.

The finding that a ketogenic diet can increase *A. muciniphila* is in contrast with a previous study, which shows that following a VLCKD resulted in lower alpha diversity and *A. muciniphila* levels [32]. It is likely that this effect is mediated by the fact that a VLCKD also has extremely reduced complex carbohydrates (i.e., fibres), which serve as a fuel source for micro-organisms, such as *A. muciniphila* [33]. It is thus important for the interpretation of the results to define the exact amounts of (complex) carbohydrates when conducting an intervention trial using a ketogenic diet.

Most human studies that investigated the effects of a ketogenic diet on epilepsy are observational in their design, and randomized controlled trials (RCTs) are still lacking. However, multiple (prospective) studies show that a ketogenic diet is effective in reducing seizures in patients that suffer from refractory epilepsy [16,21]. Interestingly, the role of the gut microbiota with regards to the anti-seizure effects of a ketogenic diet has also been established in human studies [34,35,36,37,38,39]. One study found that a ketogenic diet (4:1) reduces faecal SCFA in subjects with therapy resistant epilepsy [34]. The authors hypothesized that this decrease was due to lower levels of (complex) carbohydrates which reached the gut microbiota, and therefore led to lower levels of SCFA-producing bacteria. However, previous studies found that plasma SCFAs are better markers for metabolic health than faecal SCFA [40], and therefore this finding needs to be interpreted with caution. As written above, SCFAs are often associated with improved metabolic health [29], and also play a pivotal role in the gut–brain axis [41]. Multiple studies in epileptic patients and one study in subjects suffering from GLUT1-DS showed that a ketogenic diet intervention (4:1) can indeed alter gut microbial composition [18,36,42]. Despite the reduction of seizure frequency, gut microbiota composition showed a reduction in healthy bacteria upon a ketogenic diet. However, it is important to emphasize that these studies are to be considered as pilot studies and larger clinical trials are needed to elucidate the interaction between gut microbiota and the ketogenic diet on reducing seizure activity in (refractory) epilepsy.

## 3. The Gut Microbiota and Ketogenic Diet: Obesity

The ketogenic diet has gained popularity as a method to reduce weight, by inducing a metabolic switch towards fat oxidation which can subsequently reduce hunger by the production of ketone bodies [7,43]. Interestingly, mice that followed a ketogenic diet (78.9% fat, 9.5% protein, 0.76% carbohydrates (*wt/wt*)) had the same amount of weight loss as mice on a 66% caloric restriction after 9 weeks, whereas mice on a high-fat, high sucrose or a chow diet showed no weight loss [44]. However, this weight loss might not be sustainable as another study in mice showed, despite initial weight loss, no weight loss was observed after 22 weeks of following a ketogenic diet (72% fat, 10% protein, 2% carbohydrates (g)), indicating potential differences between short-term and long-term effects of a ketogenic diet on body weight [45]. Conversely, a systematic review with a meta-analysis showed that a ketogenic diet in obese adults is more effective than a low-fat diet for long-term (≥12 months) weight loss [46]. In addition, the review of Nabrdalik et al. (2021) suggests that the short and long-term (1–24 months) effects of a low-carbohydrate diet on weight loss is often more effective than a low-fat diet [47]. Yet, there are contrasting theories about the exact mechanisms. Possible mechanisms include lower energy intake, increased satiety due to higher protein, and low-carbohydrate consumption [48,49]. It is also possible that the gut microbiota are involved in mediating weight loss through a ketogenic diet [17]. Possible mechanisms include increased production of microbial metabolites, like SCFA, that can cross the blood–brain barrier and affect food intake [50]. As mentioned above, SCFA are mainly produced from fibres, it can therefore be confusing as to how ketogenic diets can promote the production of SCFA. One explanation would be the fact that a KD can increase SCFA production by potentially increasing the amount of SCFA producing bacteria like A. *Muciniphila.* Another explanation would be the fact that SCFA are also postulated to be able to be formed from fermentation of dietary protein [51,52,53]. However, it is important to note that these findings are mainly based on relatively old in vitro experiments and more detailed research is needed focusing on the potential of SCFA production from dietary protein.

Another interesting study in both mice and humans found that a ketogenic diet (4:1) led to decreased levels of *Bifidobacterium* [54]. This decrease was mediated through the increased production of ketone bodies, most importantly beta-hydroxy butyrate, and led to lower levels of intestinal and visceral fat pro-inflammatory Th17 cells. This is an important finding as insulin resistance and obesity are characterized by low-grade inflammation and reducing Th17 cells may help in reversing this process. However, it is also important to note that increased abundance of *Bifidobacteria* in general have been positively associated with human health and are even being used as probiotics [55,56].

To date, no preclinical studies investigated the effects of a ketogenic diet on the gut microbiota and body weight. However, there is a mouse study that investigated a high-fat diet [57]. This study showed that body weight significantly increased in specific pathogen free mice (i.e., mice that still have gut microbiota) compared to germfree mice (i.e., mice that lack microbiota) after following a cholesterol-rich high-fat diet, but not after a low-cholesterol high-fat diet (48 En% fat, 34 En% carbohydrates, and 18 En% protein in both diets). This underscores that dietary cholesterol might affect the crosstalk between gut microbiota and the host metabolism, which could drive obesity in specific pathogen free mice. Currently, three clinical studies investigated the effects of a VLCKD on the gut microbiota and body weight in humans. The single-blind RCT of Gutiérrez-Repiso et al. (2019) [58] investigated the effects of a 4-month VLCKD (ratio not given) followed by a low-caloric diet in 33 obese Spanish adults aged 18–65 years and compared a VLCKD with or without the addition of synbiotics (i.e., a combination of pre- and probiotics). The authors concluded that the VLCKD resulted in weight loss and in a beneficially altered gut microbiota composition such as an increase in microbial diversity. However, no difference in richness was observed [58]. In addition, a randomized pilot study compared the addition of whey, vegetable, or animal protein to a 45-day long VLCKD (40.4% fat, 46.1% protein, and 13.5% carbohydrates) in 48 obese Italian adults and observed weight loss in all groups [59]. The study also showed that all VLCKDs resulted in a healthier microbiota composition by a decrease in Firmicutes and an increase in Bacteroidetes, which was more pronounced in the whey and vegetable protein group. However, a randomized parallel study with 91 Australian overweight or obese adults aged 24–64 years showed that an 8-week low-calorie and low-carbohydrate (61% fat, 35% protein, and 4% carbohydrate) diet resulted in impaired bowel health (i.e., decreased stool mass, decreased bowel movement frequency, and reduced faecal butyrate concentration) compared to an isocaloric high-carbohydrate (30% fat, 24% protein, and 46% carbohydrates) diet [60]. The amount of complex carbohydrates was also higher in the latter group, which may explain the found differences. Cereal, bread, fruit, potato, pasta, rice, and beans were provided as the carbohydrates in the high-carbohydrate diet. Another detrimental effect of the low-carbohydrate diet is that the abundance of *Bifidobacteria* significantly decreased, which was not seen after following the high-carbohydrate diet. Both diets resulted in weight loss, which was greater in the low-carbohydrate group.

The beneficial effects of the ketogenic diet on weight reduction and the exact role of the gut microbiota remains elusive. Long term (i.e., >2 years) as well as medium (i.e., > 6 months–2 years) and short term (i.e., <3 months) studies are studies are needed to investigate if the induced weight loss is sustainable, preferably by also including a wash-out period and cross-over design with a “normal” baseline diet. Furthermore, weight loss itself can alter the gut microbiota composition. This makes it difficult to disentangle a clear effect of a ketogenic diet versus weight loss itself on the gut microbiota composition and function.

## 4. The Gut Microbiota and Ketogenic Diet: Dyslipidaemia

Dyslipidaemia is characterized by lipid abnormalities, i.e., increased total cholesterol, low-density lipoprotein (LDL)-cholesterol, and triglyceride plasma levels or decreased high-density lipoprotein (HDL)-cholesterol and is a major risk factor for cardiovascular disease [61]. A large clinical study and a review show that (abdominal) obesity and diabetes are common risk factors for dyslipidaemia [62,63].

As described before, a ketogenic diet can lead to weight loss, which has a beneficial impact on blood lipid levels [46,47,64]. However, results about the effects of a ketogenic diet on blood lipid levels are conflicting. Currently, only a recent mouse study investigated the effects of two commonly used ketogenic diets, a ketogenic diet type 1 (KDR) and a ketogenic diet type 2 (KDH), on fat accumulation and gut microbial profile [65]. The authors found that the KDH (91.3% fat (derived from corn oil and hydrogenated vegetable shortening, low in trans-fatty acids), 1% carbohydrates, and 7.7% protein) but not the KDR diet (89.5% fat, derived from corn oil and Primex, high in trans-fatty acids), 0.1% carbohydrates, and 10.4% protein) increased excessive lipid accumulation in the liver with increased total cholesterol and triglyceride concentrations. Despite the increased richness and diversity in the KDH, the change in microbiota composition in both the ketogenic diets was associated with lipid accumulation, mostly in the KDH. The main difference between these diets were the fat sources, which should be taken into account when investigating the potential effects of a ketogenic diet. To date, however, no clinical studies investigated the effects of a ketogenic diet on both the blood lipid levels and gut microbiota.

## 5. The Gut Microbiota and Ketogenic Diet: Insulin Resistance

Metabolic alterations and subsequent diseases caused by a Western lifestyle are commonly referred to as cardiometabolic diseases (CMD), ranging from metabolic syndrome, type 2 diabetes to cardiovascular diseases [66]. One of the key metabolic derangements in CMDs is insulin resistance as it plays a central role in obesity, metabolic syndrome, as well as type 2 diabetes [67]. Both dietary interventions, as well as gut microbiota composition and function have independently been associated with insulin resistance [68,69]. Of all macronutrients, increasing or decreasing carbohydrate intake has the strongest influence on insulin resistance and possibly the gut microbiota [33,70,71]. However, the safety and potential benefit of a ketogenic diet with regards to insulin resistance has been a point of discussion [5,45,65,72,73].

The study of Li et al. (2021) described in the section above shows how a ketogenic diet (KDR and KDH diets in this study) can exert a systemic metabolic change as it can not only affect host lipid levels, but also glucose homeostasis [65]. The authors showed that the KDR diet, but not the KDH diet increased fasting glucose levels and insulin resistance. This finding might be mediated by the increased levels of *Bacteroidetes* and an altered bile acid profile. Previous work supports this finding as transplantation of *Bacteroidetes vulgatus* to mice intestines led to increased insulin resistance [74]. Moreover, the gut microbiota alpha diversity was only increased in the KDH group, but not the KDR group, reflecting a more favourable gut microbiota composition. However, both diets differed in their fat source, which could have affected the outcome.

Clinical trials investigating the interaction between a ketogenic diet, gut microbiota composition and insulin resistance are scarce. In fact, only one clinical trial could be identified through our search, which is also described before [59]. The authors of this study investigated the effects of three different 45-day VLCKD (40.4% fat, 46.1% protein, and 13.5% carbohydrates) in 48 obese subjects with insulin resistance. The VLCKD were isocaloric but differed in their protein content (whey protein, vegetable protein, and animal protein). All three diets induced significant weight loss and improved insulin resistance. However, the VLCKD with animal protein significantly reduced renal function compared to baseline, whereas the other diet groups did not. All three diets changed the gut microbiota composition significantly, with an increase in Bacteroidetes and decrease in Firmicutes abundance. The authors found that a whey protein VLCKD and a vegetable protein VLCKD led to a greater Firmicutes abundance decrease with regards to their respective baseline, than the animal protein VLCKD group. This study was limited by the fact that the inclusion numbers were small and there was no follow-up. Therefore, the stability of the changes induced to the metabolic parameters and gut microbiota composition cannot be determined. Moreover, the subjects were obese and insulin resistant at baseline and therefore their baseline gut microbiota composition is likely disturbed at baseline and not representative for a healthy population. Results of this trial therefore need to be interpreted as pilot results and larger, better defined dietary trials are needed to study the role of a ketogenic diet on gut microbiota composition/function and metabolic health.

SCFA, short-chain fatty acids; dashed arrows mean low certainty, full arrows mean high certainty. A ketogenic diet affects the gut microbiota and with it also multiple domains of human health. A ketogenic diet increases the alpha diversity of the gut microbiota and affects the Firmicutes/Bacteroidetes ratio. Moreover, a ketogenic diet is associated with decreases in fecal SCFA and increases in *A. muciniphila*. These markers are associated with improved metabolic health and are associated with less seizure activity, increased insulin sensitivity and higher weight loss.

## 6. Summary and Future Perspectives

**Figure 1:**
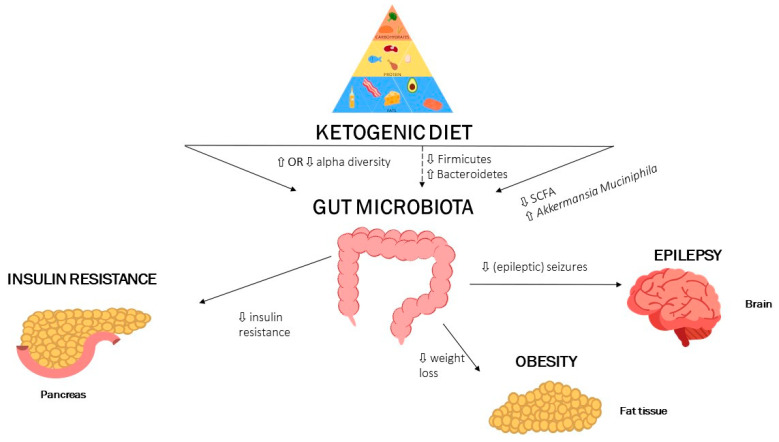
Summary findings: A ketogenic diet influences alpha diversity, but studies are conflicted whether is increases or decreases alpha diversity.

In recent years the ketogenic diet is gaining popularity as a method to promote weight loss and improve cardiometabolic disease (CMD) [7,17,49]. The ketogenic diet has been shown to influence multiple domains of metabolic health, such as obesity, insulin resistance, dyslipidaemia, and its original source of popularity, reducing seizures [1,7,37]. Interestingly, the gut microbiota has been implicated in all these factors and likely plays a crucial role in mediating, at least in part, the beneficial effects of a ketogenic diet on metabolic health. These findings have been summarized in Figure 1.

It is important to note that the interaction between dietary interventions (such as the Mediterranean Diet) and the gut microbiota on CMD have been investigated with interesting findings [75,76]. A comparison between these effects and the specific effects of a ketogenic diet on gut microbiota modulation is interesting, but beyond the scope of this review. Especially, since clinical studies are lacking that study the interaction between a ketogenic diet, gut microbiota and CMD.

In the past, the ketogenic diet has shown significant success and gained favourable attention due to its potential to reduce (epileptic) seizures, making it the treatment of choice for patients with rare genetic mutations that can induce seizures [1,23]. However, in a recent study it was also shown that a high fat, low carbohydrate ketogenic diet has the potential to induce and increase cognitive impairment in mice [77]. The authors found that a KD increased cognitive impairment following hypoxia in mice and that this finding was associated with a gut microbiota enriched in *Bilophila wadsworthia.* Moreover, increasing the *B.wadsworthia* species was also associated with disturbed hippocampal physiology in this study.

In a landmark study in mice, Olson et al. (2018) showed that a ketogenic diet decreased gut microbial alpha diversity, but increased specific taxa like *A. muciniphila* [18]. Moreover, the ketogenic diet altered blood and brain metabolome, yielding increased GABA/glutamate amino acids in the brain. These changes led to protection against corneal induced seizures and also reduced spontaneous seizures in *KcNA1^-/-^* mice. Moreover, the authors showed that the seizure protective effects of the ketogenic diet were mediated via the gut microbiota as germ-free mice and antibiotic-treated mice had increased seizure activities, despite following a ketogenic diet [18]. Findings that a ketogenic diet reduces alpha diversity are in contrast with other studies [58,65]. However, beneficial species like *A. muciniphila* were increased, indicating that overall diversity might be less important than increase in specific taxa. How *A. muciniphila* can have seizure lowering effects is still unclear, but it has been suggested that an increased capacity to form microbially-produced beneficial metabolites such as SCFA which might play an important role as they can cross the blood–brain barrier and affect neural functioning [17].

To date, no clinical trial has been performed which investigates the effects of a ketogenic diet on gut microbiota composition and function in patients suffering epileptic seizures. Such a study, with an antibiotic arm, is warranted to elucidate the causal role of the gut microbiota in the seizure diminishing effects of the ketogenic diet.

The ketogenic diet is popular due to its potential to induce rapid weight loss [7,78], which can also affect gut microbiota composition and function [79]. Thus far, large dietary clinical trials in humans and also experiments in rodents are lacking that properly investigate the effect of a ketogenic diet on weight loss and gut microbiota composition and function. A recent Spanish randomized-controlled trial in 33 obese patients did report that following a VLCKD for four months has the potential to induce rapid weight loss [34]. Moreover, this diet was accompanied by an increase in gut microbiota diversity and a reduction in Proteobacteria with an increase in the *Firmicutes* phyla. These changes were more pronounced in the study group that received a symbiotic (*B. animalis subsp. lactis* and prebiotic fiber). The addition of synbiotics to the VLCKD did not alter its beneficial effects on gut microbiota diversity. The effects of a long-term ketogenic diet on obesity, metabolic health, and gut microbiota composition and function remains to be studied.

The ketogenic diet has also been studied with regards to dyslipidaemia, which affect (cardiovascular) morbidity and mortality [80]. The gut microbiota has been implicated in modulating dyslipidaemia and thereby also cardiovascular health [66]. However, currently there are no clinical studies that have studied the effects of a ketogenic diet on dyslipidaemia and gut microbiota composition and/or function. Rodent studies have shown conflicting results reporting that a ketogenic diet can increase lipid levels, while still favourably altering the gut microbiota composition [65].

Insulin resistance is one of the hallmarks of metabolic derangement and plays a crucial role in cardio-metabolic morbidity and mortality [67]. The gut microbiota has been heavily implicated in its potential to alter insulin resistance [81]. However, to date only one study has investigated the role of a ketogenic diet on insulin resistance, taking gut microbiota into account [59]. This Italian pilot clinical trial investigated the effects of several isocaloric 45-day VLCKD on metabolic health parameters in 48 obese subjects. The authors found that a VLCKD, regardless of whey, plant, or animal protein supplementation, improved insulin sensitivity and decreased body mass index (BMI). The authors also found that this improvement was accompanied by a decrease in *Firmicutes*, but increase in *Bacteroidetes* phyla. Consumption of animal protein with a VLCKD did result in less beneficial effects as the *Firmicutes* and *Bacteroidetes* changes were less pronounced, and subjects had higher markers of renal damage. These results favour a VLCKD with an emphasis on mainly plant protein sources.

## 7. Limitations

Several limitations have to be considered for this work. First, a ketogenic diet is a general description with multiple definitions of diets that can induce ketosis (e.g., medium-chain triglyceride diet, Low Glycaemic Index treatment, The Modified Atkins Diet). Each diet differs in macronutrient composition and can thus have different metabolic effects. In order to identify all relevant studies that looked at the combined effect of a ketogenic diet and gut microbiota with regards to metabolic health we performed a systemic search strategy (Supplementary Table S1) and found that most studies were done using the “classical” or VLCKD. It is therefore important to emphasize that studies using different dietary protocols, but can still induce ketosis, may give different findings. These studies are warranted in order to put the effect of a ketogenic diet and the gut microbiota into a broader perspective.

Second, large clinical randomized trials are still lacking that study the effects of a ketogenic diet on gut microbiota composition and function with regards to metabolic health. Human data that support rodent findings are mainly small pilot clinical trials with poorly defined gut microbial outcomes and analysis methods. These trials are needed in different populations (i.e., healthy, obese, diabetic, or epileptic patients) in order to gain more insight in the (patho)physiological response of a ketogenic diet on gut microbiota and metabolic health. Currently, multiple clinical trials are ongoing studying the effects of a ketogenic diet in non-alcoholic fatty livers disease (clinicaltrials.gov registration: NCT03784716), Type 2 diabetes (clinicaltrials.gov registration NCT04791787) and obesity with/without type 2 diabetes (clinicaltrials.gov registration NCT05071287). Of these limited number of trials ongoing, only NCT05071287 has registered to also study the role of the gut microbiota.

Third, due to the heterogeneity in preclinical work, and the lack of well performed clinical trials it is currently not possible to provide a “main” mechanism of mechanisms by which a ketogenic diet influences metabolism as a whole. We believe that a ketogenic diet has the potential to alter the gut microbiota composition and function, thereby promoting alpha diversity but also the production of beneficial microbial metabolites. This in turn plays a role in the beneficial effects of a ketogenic diet on seizure activity, obesity, dyslipidaemia, and insulin resistance. However, more work is needed to put this hypothesis into context and to be able to provide more detailed mechanistic theories. Moreover, due to the above-mentioned limitations it is also not possible at this moment to provide clear, “main” microbiota changes other than the fact that a ketogenic diet alters alpha diversity and the production of microbially produced metabolites.

## 8. Conclusions

Multiple studies have shown the beneficial effects of a ketogenic diet on metabolic health and reduced seizure activities. These effects are likely, at least in part, mediated via the gut microbiota. However, in all domains large clinical trials are lacking incorporating a ketogenic diet, gut microbiota, and metabolic health. To date, most evidence comes from rodent research and small clinical trials. Clinical trials and prospective cohorts are needed to elucidate the mediating role of the gut microbiota in the beneficial metabolic effects of a ketogenic diet. This will give rise to potentially altering the gut microbiota prior/during a ketogenic diet using pre-, pro-, or synbiotics which can improve the effectiveness of the ketogenic diet.

## Supplementary Materials

The following are available online at https://www.mdpi.com/article/10.3390/nu14010191/s1, Table S1: Search terms.

## Abbreviations

BMI	Body mass index
CMD	Cardiometabolic diseases
En%	Energy percentage
GLUT1-DS	Glucose Transporter-1Deficiency syndrome
SCFA	Short-chain fatty acid
VLCKD	Very low-caloric ketogenic diet
LDL	Low-density lipoprotein
HDL	High-density lipoprotein
