# Supplementary material for: The Role of the Gut Microbiota on the Beneficial Effects of Ketogenic Diets
Source: Nutrients. 2021 Dec 31;14(1):191. doi: 10.3390/nu14010191 (PMC8747023; doi:10.3390/nu14010191)
Supplement: Supplementary file 1 [file nutrients-14-00191-s001.zip › nutrients-1477772-supplementary.pdf]

Table S1. Search terms.

**Search terms**
Obesity
((((ketogenic diet[tiab]) OR (ketogenic diet[mesh])) OR (low carbohydrate diet)) AND ((gut microbiota[mesh]) OR (gut microbiot*[tiab]))) AND (central adiposity OR obesity OR overweight OR body composition OR weight-loss)
Dyslipidemia
((((ketogenic diet[tiab]) OR (ketogenic diet[mesh])) OR (low carbohydrate diet)) AND ((gut microbiota[mesh]) OR (gut microbiot*[tiab]))) AND (dyslipidemia OR cholesterol OR LDL OR VLDL OR HDL)
insulin resistance
((((ketogenic diet[tiab]) OR (ketogenic diet[mesh])) OR (low carbohydrate diet)) AND ((gut microbiota[mesh]) OR (gut microbiot*[tiab]))) AND (insulin OR insulin resistance OR glucose OR diabetes)
seizure
((((ketogenic diet[tiab]) OR (ketogenic diet[mesh])) OR (low carbohydrate diet)) AND ((gut microbiota[mesh]) OR (gut microbiot*[tiab]))) AND (seizure OR epilepsia))
NB: searches were also combined
